# The most important components of out-of-hours community care for patients at the end of life: A Delphi study of healthcare professionals’ and patient and family carers’ perspectives

**DOI:** 10.1177/02692163221106284

**Published:** 2022-06-29

**Authors:** Joanna Goodrich, Lydia Tutt, Alice M Firth, Catherine J Evans, Fliss EM Murtagh, Richard Harding

**Affiliations:** 1 Florence Nightingale Faculty of Nursing Midwifery a Palliative Care, Cicely Saunders Institute, King’s College London, London, UK; 2 Wolfson Palliative Care Research Centre, Hull York Medical School, University of Hull, Hull, UK

**Keywords:** Palliative care, community, after-hours care, Delphi technique

## Abstract

**Background:**

Community services for palliative patients outside normal working hours are variable and the best evidence-based models of care have not been determined.

**Aim:**

To establish expert consensus on the most important components of out-of-hours community palliative care services.

**Design:**

Delphi study. The first round listed 68 components generated from systematic literature reviewing, focus groups with healthcare professionals and input from the project’s patient and public involvement advisory group. The components deemed ‘essential’ by over 70% of participants in the first round were refined and carried forward to a second round, asking participants to rank each on a five-point Likert scale (5 highest to 1 lowest). The consensus threshold was median of 4 to 5 and interquartile range of ⩽1.

**Participants:**

Community specialist palliative care health professionals, generalist community health professionals and patients and family carers with experience of receiving care out-of-hours at home.

**Results:**

Fifty-four participants completed round 1, and 44 round 2. Forty-five components met the threshold as most important for providing out-of-hours care, with highest consensus for: prescription, delivery and administration of medicines; district and community nurse visits; and shared electronic patient records and advance care plans.

**Conclusions:**

The Delphi method identified the most important components to provide community palliative care for patients out-of-hours, which are often provided by non-specialist palliative care professionals. The importance placed on the integration and co-ordination with specialist palliative care through shared electronic records and advance care plans demonstrates the reassurance for patients and families of being known to out-of-hours services.

**What is already known about the topic?**

- The majority of people say that they would prefer to be cared for at the end of their lives at home, and die at home.
- The number of home deaths has been increasing, even more so during the Covid-19 pandemic.
- Community services for palliative patients outside normal working hours are variable and there is no consensus about optimal models of care.

**What this paper adds**

- There is consensus regarding the components most important for patients and families being cared for at home. These are often provided by non-specialist palliative care professionals (specifically general practitioners (GPs), district and community nurses, and pharmacists).
- Electronic patient records and advance care plans shared between healthcare providers are rated as of highest importance, demonstrating the priority for patients and families of being known to out-of-hours providers for continuity of care.
- The Delphi method enabled those with experience of providing and receiving out-of-hours care to establish consensus.

***Implications for practice, theory or policy***

- The most important components of out-of-hours community palliative care have been identified by the participants in this study, making a valuable contribution to the knowledge base which should inform the commissioning of services.
- Components reaching the highest consensus were prescribing, delivery and administration of medicines; clinical nursing care by district and community nurses; and shared electronic patient records and advance care plans for integrated working and care coordination across healthcare providers.
- Findings provide new evidence to inform the design of models of out-of-hours care to be developed and tested through further research.

## Background

The majority of people would prefer to be cared for, and die, at home at the end of life.^1^ Global demand for community palliative care is increasing.^2,3^ A cross-national study showed that home and nursing home were the two places of death with the highest palliative care needs.^4^ However, home death rates differ between countries due to inequalities of access to palliative care.^5,6^ During the Covid-19 pandemic in 2020, the number of home deaths increased in the UK.^7^ In England and Wales for example, the number increased to one-third higher than in the previous 5 years (from 125,000 to 167,000)^8^ and there was a 77% increase in home deaths in the 11 weeks between 7th March 2020 (*n* = 2725) and 15th May 2020 (*n* = 4834).^9^ Internationally there is limited data available on home deaths during the Covid-19 pandemic, but it is known that, as in the UK where an increased demand has been placed on community and primary care services,^10^ a substantial proportion in other countries have been among care home residents.^11^

‘Out-of-hours’ care comprises care provided in the evenings, overnight, weekends and public holidays and provision is variable.^12^ In the UK delivery is by non-specialists where palliative care is part of their role, such as general practitioners (GPs) and community nurses, and specialists in palliative care where palliative care is all of their role, such as consultants in palliative medicine and clinical nurse specialists.^13^ There are recurrent problems in the provision of out-of-hours care, including the challenge of co-ordinating care between different services, uncertainties around prognostication and decision-making about admission to hospital, while taking into account the capacity to care (by informal and formal carers) for the patient at home and the wishes of patient and family.^14,15^ To date there is a lack of agreement about which models of care (and which components of these models), including out-of-hours provision, provide good quality care for patients and families near end of life.^16^

## Method

*Research question*: The aim of this study was to establish expert consensus on the most important service components required for high-quality, out-of-hours palliative care for patients in the community.

*Design*: Delphi methods were utilised to establish expert consensus while giving weight to all voices and allowing iteration and refinement between rounds.^17,18^ A two-round Delphi survey was planned, with an optional third round if consensus was not reached, reported using CREDES (Conducting and REporting DElphi Studies).^19^

Survey questions were derived from three sources: (1) findings from a systematic literature review^20^ that identified components of out-of-hours service provision, characterised in terms of *what* is provided, *when* and *by whom*; (2) findings from two focus groups held in 2020 and 2021 with community specialist palliative care professionals (who were not participants in the Delphi study); (3) recommendations from an external partner workshop to determine priorities for out-of-hours community palliative care.^21^ Members of the study’s patient and public involvement (PPI) advisory group met with researchers to review and refine questions to consider priorities for patients and families and accessibility of language, and to help interpret results. An external pilot was then conducted with two palliative care clinicians (non-participants) and three PPI advisory group members. The surveys were developed and administered using an online survey tool (http://www.onlinesurveys.ac.uk). Each round was open for 2 weeks (round 1 April 2021, round 2 May 2021); a reminder email was sent in the second week. The a priori criterion to conduct a third round was if consensus (70%) was not reached in Round 2 for at least five components.

*Setting*: The questions in the Delphi study were related to care provided in the UK to palliative patients at home (outside normal working hours), either by specialist palliative care community services or by non-specialist community and primary care services.

*Population*: Multidisciplinary specialists and non-specialists in palliative care providing care to patients in the community (palliative care medical consultants and specialist nurses, district and community nurses, healthcare assistants and general practitioners). Adult patients or family members with experience of receiving care at home outside normal working hours.

*Sample*: A selective sampling procedure was followed^18^; participants were selected with the purpose of applying their knowledge to the study. The criterion for selection was experience in either delivering care to palliative patients at home out-of-hours, or receiving care at home out-of-hours.

*Recruitment*: participant*s* were recruited via email invitation, which included information about the study, through the nine specialist palliative care sites (charitable hospices and NHS specialist teams) in England and Scotland enrolled in the study. Invitations were cascaded to colleagues in Northern Ireland and Wales. Participants were asked to invite their community and primary care partners to take part. Adult patients or family members were recruited through advertisement on two Patient and Public Involvement (PPI) forums whose members are affected by serious illness as carers or patients (These participants will henceforth be referred to as ‘patient/family participants’).

*Data collection Round 1*: For the list of 68 components, participants stated whether each was provided by their service (or, for patient/family participants, had been received), and ranked its importance for out-of-hours care (‘essential’, ‘desirable’ or ‘not at all’). Responses distinguished between evenings and overnight, weekends and public holidays, with opportunity to add free text comment.

*Data collection Round 2*: For the retained 46 components, participants were asked to delineate components further by ranking them on a 5-point Likert scale (1 = ‘not at all important’, 2 = ‘limited importance’, 3 = ‘neutral, 4 = ‘important’ 5 = ‘extremely important’) again with opportunity to add comments.

*Data analysis Round 1*: In order to reflect potentially divergent views of both patient/family and healthcare professionals, components were retained if 70% of *either* group agreed the component was ‘essential’. Descriptive statistics (frequencies) were calculated using SPSS. Free text comments were used to refine the design of phase 2, and some questions were reworded where there was a suggestion that language needed to be more accessible (in relation to medication e.g.).

*Data analysis Round 2*: Responses were analysed using SPSS to identify components with an interquartile range (IQR) of 1 or less and median = 4–5. Components with strongest consensus had IQR of 0 and median = 5. The a priori criterion for conducting a third round was not met.

*Ethical issues*: This Delphi study is part of a wider study, ‘Understanding and improving out-of-hours community palliative care’ for which NHS Health Research Authority ethical approval was received (London-Bloomsbury REC 19/LO/1865).

A statement at the start of the survey indicated that completion would be taken to assume consent to use the data.

## Results

### Round one

Fifty-four individuals participated (25 specialist healthcare professionals, 16 non-specialist healthcare professionals and 13 patient/family) mainly from England (*n* = 46), Wales and Scotland (see Table 1). The healthcare professionals were specialists in palliative care (*n* = 25) or non-specialist (*n* = 16). All were experienced in providing palliative care (mean 12.8, range 1–34 years). Most professionals identified community services as their main work setting for palliative care provided by both hospices (*n* = 28) and the NHS (*n* = 10).

**Table 1.** Role and locations of participants by country.

Role	England	Wales	Scotland	Undisclosed	Total
Palliative care medical consultant	4	1	0	1	6
Palliative care medical registrar	1	0	0	0	1
Palliative care clinical nurse specialist	15	0	2	1	18
General practitioner	4	0	0	0	4
District nurse	2	0	1	0	3
Other professional	9	0	0	0	9
Patient/family participant	11	0	2	0	13
Total	46	1	5	2	54

Table 2 shows all the components for out-of-hours community palliative care services, with proportions shown for the ‘essential’ category, presented as an overall score and according to role (patient/family and healthcare professional).

**Table 2.** Round 1: Service components for out-of-hours palliative care services in the community with proportions who considered component ‘essential’.

Service component	Proportion who considered the component essential			Included in Round 2
	Patient/family (*n* = 13)(%)	HCPs (*n* = 41)(%)	Combined (*n* = 54) (%)	
**1. Telephone advice for patients and carers**				
Single telephone number	76.9	65.9	68.5	Y
National telephone number for patients and carers	46.2	20.5	26.9	N
Local telephone number for patients and carers	69.2	85.0	81.1	Y
Same telephone number for ‘in-hours’ and ‘out-of-hours’	53.8	65.0	62.3	N
Dedicated telephone line for PC and EOL only	69.2	73.2	72.2	Y
HCPs sharing direct telephone numbers	15.4	30.0	26.4	N
Person on phone is trained in communicating with palliative care patients and carers	84.6	95.1	92.6	Y
Person on phone has quick access to patient’s medical notes or summary	91.7	85.4	86.8	Y
**2. Medicines management**				
Being able to speak to a prescriber, such as a GP or nurse, when needed	84.6	82.9	83.3	Y
Getting a visit from prescriber, such as a GP or nurse, when needed	69.2	87.8	83.3	Y
Being prescribed anticipatory medicines	69.2	97.6	90.7	Y
A pharmacy open out-of-hours locally	46.2	92.7	81.5	Y
Being able to have medicines delivered in the evenings and on weekends	53.8	65.0	62.3	Y*
Visits from a healthcare professional who can give medicines such as injections when needed	69.2	100.0	92.3	Y
**3. Crisis support**				
Visit from a GP	54.5	72.5	68.6	Y
Telephone or video consultation with a GP	58.3	65.8	64.0	N
Visit from a specialist palliative care doctor	50.0	40.0	42.3	N
Telephone or video consultation with a specialist palliative care doctor	66.7	53.8	56.9	N
Visit from a specialist palliative care nurse	75.0	63.2	66.0	Y
Telephone or video consultation with a specialist palliative care nurse	66.7	79.5	76.5	Y
Rapid response nursing service provided by community nurses	83.3	84.6	84.3	Y
Paramedics trained to provide palliative care support	63.6	65.0	64.7	N
Direct referral to hospice bed when needed and requested	60.0	52.5	54.0	Y*
Direct referral to hospital bed when needed and requested	63.6	57.9	59.2	Y*
Managing blocked or leaking (urinary) catheters	76.9	95.1	90.7	Y
Preventing or managing pressure sores	76.9	72.5	73.6	Y
Providing urgent equipment such as commodes, bed pans or beds	69.2	65.9	66.7	N
Urgent support with nutrition and hydration	69.2	36.6	44.4	N
Urgent (same day) increase in visits from usual carers	46.2	73.2	66.7	Y
Support when complex acute symptoms develop, such as pain or breathlessness	84.6	97.5	94.3	Y
Emotional support for patients	46.2	72.5	66.0	Y
**4. Supporting families and carers**				
Dedicated advice line for supporting carers and families	53.8	68.3	64.8	N
Urgent respite admission (when a carer is not well, e.g.)	69.2	42.5	49.1	N
Urgent support from a community or volunteer service	23.1	40.5	36.0	N
Being involved in care decisions taken out-of-hours, for example, admission to hospital	66.7	71.8	70.6	Y
Being involved ahead of time in making advance care plans	69.2	79.5	76.9	Y
Providing the family with information about what to do in a crisis	84.6	92.5	90.6	Y
Emotional support for family	38.5	85.4	74.1	Y
Bereavement support for family	46.2	70.7	64.8	Y
**5. Considering the last few days of life**				
Home visits from GP	53.8	74.4	69.2	Y
Home visits from a district or community nurse	76.9	100.0	94.1	Y
Home visits from a specialist palliative care nurse	69.2	71.8	71.2	Y
Home visits from a specialist palliative care doctor	53.8	43.6	46.2	N
Rapid response nursing service	76.9	81.6	80.4	Y
Night sitting service	66.7	81.1	77.6	Y
Personal care (e.g. help with washing)	61.5	81.6	76.5	Y
Being able to get medicines prescribed at short notice	76.9	89.5	86.3	Y
Being able to get medicines delivered at short notice	72.7	71.8	72.0	Y
Being able to get medicines administered at short notice	69.2	97.4	90.4	Y
Existential or spiritual support	38.5	46.2	44.2	N
Having an advance care plan in place and regularly reviewed with patient and family	46.2	74.4	67.3	Y
Having access to any equipment needed	75.0	67.5	69.2	Y
Urgent help with hydration	83.3	33.3	45.8	Y
**6. During and immediately following death**				
Rapid confirmation of death	84.6	71.8	75.0	Y
Ability to confirm or verify death via telephone or video link	46.2	18.4	25.5	N
Rapid access to undertakers	46.2	82.1	73.1	Y
Availability of culturally relevant information and support for families	41.7	66.7	60.8	N
Availability of culturally relevant information and support for healthcare providers	33.3	57.9	52.0	N
**7. Continuity of care and integrating services**				
Specialist telephone advice for healthcare providers	66.7	90.0	84.6	Y
Specialist telephone advice for social care providers	66.7	65.0	65.4	N
Knowledge among healthcare providers about the different out-of-hours services and roles	75.0	82.5	80.8	Y
Direct referral between services (not just signposting patients)	90.9	71.8	76.0	Y
Patients keeping a physical copy of their medical notes or summary for any at-home visits	75.0	36.8	46.0	Y
Shared electronic patient records between all services (specialist, generalist, night and day), for example, EPaCCS^22^	83.3	85.0	84.6	Y
Advance care plans shared between services (e.g. using national ReSPECT document)^23^	75.0	77.5	76.9	Y
Having a palliative care co-ordination hub to oversee local service integration	54.5	45.9	47.9	N
Different services co-located together in one place	63.6	24.3	33.3	N
Having the same service available 24/7 (no difference between out-of-hours and ‘office hours’ services, but could include reduced staffing)	58.3	59.5	59.2	N

* Component did not reach agreed consensus but went on to be included in Round 2 because free text responses suggested that there had been confusion about meaning.

PC: palliative care; EOL: end of life; HCP: healthcare practitioner; GP: general practitioner.

### Round Two

Of *N* = 54 round one participants, *n* = 48 agreed to be contacted for Round 2 and of these *n* = 44 (91.6%) completed round 2 (11 patient/family and 33 healthcare professionals, 24 specialists and 9 non-specialists Round 2 re-presented 46 items and 98% reached the agreed threshold for consensus (see Table 3).

**Table 3.** Round 2: Consensus on the important components for out-of-hours palliative care services in the community.

If you were to design an out-of-hours community palliative care service, please rate how important you think these components would be:	Median score	IQR
1. Telephone advice for patients and carers		
Person on phone has quick access to patient’s medical notes or summary*	5	0.00
A single telephone number	5	1
Dedicated telephone line for palliative care and end of life patients only	5	1
Person on phone is trained in communicating with palliative patients and carers	5	1
Local telephone number	4	1
2. Medicines management		
Visits from a healthcare professional who can give medicines such as injections when needed*	5	0.00
Being provided with anticipatory medicines*	5	0.75
Being able to speak to a prescriber, such as a GP (general practitioner) or nurse, when needed	5	1
Getting a visit from a prescriber, such as a GP or nurse, when needed	5	1
Being able to have medicines delivered in the evenings and weekends*	5	1
A pharmacy being opened out-of-hours locally	5	1
3. Crisis support		
Support when complex acute symptoms develop, such as pain or breathlessness*	5	0.75
Direct admission to hospice bed when needed	5	1
Rapid response nursing service provided by community nurses	5	1
Managing blocked or leaking (urinary) catheters	5	1
Urgent (same day) increase in visits from usual carers	5	1
Visit from a GP	4	1
Visit from a specialist palliative care nurse	4	1
Telephone or video consultation with a specialist palliative care nurse	4	1
Direct admission to hospital bed when needed	4	1
Preventing or managing pressure sores	4	1
Emotional support for patients	4	1
4. Family and carers		
Providing the family with information about what to do in a crisis*	5	0.00
Being involved ahead of time in making advance care plans	5	1
Emotional support for family	4	1
5. Last few days of life		
Home visits from a district or community nurse*	5	0.00
Being able to get medicines prescribed at short notice*	5	0.00
Being able to get medicines delivered at short notice*	5	0.00
Being able to get medicines administered at short notice*	5	0.00
Home visits from a specialist palliative care nurse	5	1
Rapid response nursing service	5	1
Night sitting service	5	1
Personal care (e.g. help with washing)	5	1
Having an advance care plan in place and regularly reviewed with patient and family	5	1
Having access to any equipment needed	5	1
Home visits from GP	4.5	1
Urgent help with hydration	4	1
6. During and immediately following death		
Rapid confirmation of death	4	1
Rapid access to undertakers	4	1
7. Continuity of care and service integration		
Advance care plans shared between services (e.g.in England or Scotland using ReSPECT document^23^)*	5	0.50
Shared electronic patient records between all services (e.g. specialist, generalist, night and day), for example, EPaCCS in England^22^*	5	0.75
Specialist telephone advice for healthcare professionals	5	1
Knowledge among healthcare providers about the different out-of-hours services and roles	5	1
Direct referral between services (not just signposting patients)	4.5	1

* Highest consensus.

Of the 46 Round 2 components, only one, ‘patients keeping a physical copy of their medical notes or summary for any at-home visits’ was not considered important and did not reach the threshold (median = 4, IQR = 2).

## Discussion

*What this study adds with reference to international literature*: Many countries have implemented policy measures to reduce the number of acute care hospitalisations and increase home deaths.^24,25^ Globally the need for palliative care is increasing.^4^This Delphi has identified the most important components of community-based out-of-hours palliative care, where until now there had been no consensus,^16^ and highlighted that these are provided by both specialist and non-specialist healthcare professionals (e.g. district and community nurses and pharmacists). Importantly, there was considerable agreement between healthcare professionals and patient/family participants.

*Implications for practice*: Hands-on clinical nursing care by community and district nurses in the last few days of life reached a higher consensus in round 1 (94.1% deemed this ‘essential’) than home visits by specialist palliative care nurses, specialist palliative care doctors and GPs (71.2%, 46.2%, and 69.2% respectively). In round 2, a visit from a district or community nurse reached slightly higher consensus than a visit from a specialist palliative care nurse, or a GP, both in a crisis and in the last few days of life. Findings have resource implications for all out-of-hours services, especially district and community nursing and primary care where there has been a decline in numbers particularly senior grades of community and district nurses.^26^

For our participants, timely availability of medicines is a key priority especially in the last few days of life (prescribing, delivering and administering) alongside relief of symptoms such as breathlessness and pain. These findings have implications for the commissioning of out-of-hours pharmacy provision which need careful consideration in light of the lack of evidence on the clinical effectiveness, cost effectiveness and impact on patient experience regarding key end of life medication interventions.^27^

Shared electronic records between services, or summary records being quickly available were also ranked as highly important. This reflects the global consensus between high-, middle-, and low- income countries on the need for practical approaches (particularly related to information and communication technologies) to strengthen integrated care for older people between services, including rehabilitation and palliative community services.^28^ Research in Sweden has shown how high-quality coordination of services out of hours enables patients and families to feel reassured and supported.^29,30^ Our data confirm previous qualitative findings which describe the importance of being known to out-of-hours services to palliative patients and families.^31,32^

Findings and implications were presented and discussed with both the study steering group and our PPI advisory group, and will inform the interview schedule for the next phase of the research.

*Strengths and weaknesses/limitations of the study.* This study was conducted in the UK and identifies the priority components of out-of-hours community palliative care services. However, this study may need to be repeated in other countries to understand country specific or cultural differences.

Detailed demographic data were not collected from participants and this was a missed opportunity. Although the data reflect the experience of healthcare professionals and patients and family carers, it is a limitation of the methodology that ‘outcomes are only as good as the experts and the available evidence’^19^ (p.701) and findings could differ with another sample. In particular it would be beneficial to survey a broader range of healthcare professionals who provide care out-of-hours to patients at home; this sample included predominantly specialist palliative care professionals. The next stage of the wider research study will provide the perspective of palliative patients and family members with experience of receiving care at home out-of-hours through qualitative interviews. This Delphi was conducted during the Covid-19 pandemic. Our PPI advisory group felt that responses from patient and family participants potentially reflected lower demands of services given these circumstances. This though may have focussed responses even more strongly on the core necessary components. This will be explored further in the next phase of our research.

## Conclusion

The Delphi method proved useful to generate practical and specific findings: a list of components that experts view as most important for providing out-of-hours palliative care in the community. This new evidence will be incorporated when selecting models of out-of-hours care in the next phase of the study, which will be tested further with patients and families.
